# Effect of Ball-Milled Steatite Powder on the Latent Heat Energy Storage Properties and Heat Charging–Discharging Periods of Paraffin Wax as Phase Change Material

**DOI:** 10.3390/mi13091456

**Published:** 2022-09-02

**Authors:** Sathiyalingam Kannaiyan, Song-Jeng Huang, David Rathnaraj, S. A. Srinivasan

**Affiliations:** 1 Department of Mechanical Engineering, National Taiwan University of Science and Technology, No. 43, Section 4, Keelung Rd, Da’an District, Taipei 10607, Taiwan; 2 Department of Aeronautical Engineering, Sri Ramakrishna Engineering College, Coimbatore 641022, India; 3 High Energy Batteries India Limited, Mathur 622515, India

**Keywords:** phase change material (PCMs), thermal storage system, steatite powder, energy material, solar energy, energy storage

## Abstract

Phase change materials (PCMs) serve as an advantage in thermal energy storage systems utilizing the available sensible and latent heat. The PCMs absorb the thermal energy during the charging process and release it into the environment during the discharging process. Steatite is low cost and eco-friendly, with a thermal stability up to 1000 °C, and it is abundantly available in nature. This study investigates the steatite–paraffin wax-based PCM and the effect on the cyclic loads using a horizontal triplex-tube latent heat energy storage system. The thermal conductivity value of the milled steatite-based PCM composite was 7.7% higher than pure PCM. The PCM with the ball-milled steatite-fabricated composite exhibited better discharging characteristics, increasing the discharge time by 50% more than that of the pure paraffin wax. Moreover, the milled steatite-based PCM outperformed that incorporated with non-milled steatite with paraffin.

## 1. Introduction

Energy demand is increasing exponentially every year due to the increase in globalization. The exploration of alternative energy sources is necessary. In order to achieve long-term sustainability, an appropriate quantity of energy resources must be accessible at an affordable rate and able to be used for all essential activities without negatively influencing society as per SDG goal 7 [1]. Solar energy sources are typically thought to be sustainable, since they are renewable; therefore, they can be used for many years. Although the energy available from these resources is abundant, their delivery is not consistent throughout the day or year [2,3]. This has paved the way for the development of thermal energy storage (TES) systems, which charge when power is available and discharge during its non-availability. Early research explorations in TES provided substantial supporting results. That is, the performance of the latent heat thermal energy systems (LHTES) was enhanced compared with that of the sensible heat thermal energy systems (SHTES). The reason is that LHTES has a higher capability to utilize the latent heat, a high enthalpy change during the phase change process, and compactness [4,5,6]

Phase change materials (PCMs) serve as a boon to the LHTES, which utilize the energy available to attain the phase change and store and release it during the non-availability of energy [7,8]. Among several PCMs available, organic-based PCMs are preferred because of their widespread availability, chemically stable characteristics, and non-corrosive nature. Paraffin wax is one of the organic PCMs explored for its energy storage capability in past recent decade. It melts congruently with little supercooling. Although organic paraffin PCM possesses several advantages, the thermal conductivity was in the range of 0.2 to 0.5 W/mK. To overcome this major limitation, a few investigations examined the performance with the encapsulation of second-phase particles, enhancing the overall thermal conductivity of the material. The encapsulation method provided a better enhancement, increasing the thermal conductivity compared with the embedded method as it reduced the heat conduction distances [9,10,11].

Among the two major configurations of LHTES, the horizontal LHTES showed better performance than the vertical LHTES. Moreover, among several methods to enhance the overall energy available, the change in the construction geometry was one simple way to deal with it [12,13,14]. Researchers experimented with paraffin PCM on an LHTES with fins and reported that that the extended surfaces had no significant effect on the convective heat transfer. Moreover, a 40% reduction was observed in the solidification time during conduction [15]. A similar investigation on the effect of extended surfaces was also conducted. The results substantiated that the extended surface area provided by eight fins decreased the charging time by 35% [16]. The performance of erythritol PCM was experimented on a concentric tube heat exchanger. The charging and discharging of erythritol in a concentric tube PCM with a longitudinal finned system showed better performance with insignificant sub-cooling during discharge and achieved the best charging [17].

Similarly, alumina nanoparticle-encapsulated paraffin PCM was numerically investigated under various loading conditions. The PCM with 8% alumina nanoparticles exhibited better performance in reducing the solidification time, with conduction being the dominant heat transfer mode [18]. The performance of the TES system was examined using paraffin-infused copper foam. The PCM charging rate was influenced by the inlet temperature and mass flow of the heat transfer fluid (HTF). In the presence of the copper foam in the paraffin, the PCM’s thermal conductivity was increased, and consequently, its heat transfer rate was increased [19]. Researchers investigated different types of coolants, such as PCM and nanofluid zinc oxide/water. The results showed that the PV systems’ temperature decreased by approximately 10 °C, whereas the PVT/nanofluid/PCM systems’ temperature decreased by approximately 16 °C [20].

Thermal energy can be stored most effectively with paraffin. Experimental investigations on paraffin–nano-magnetite composites indicate an increase in thermal conductivity by 48% and 67%, corresponding to 10% and 20% nano-magnetite particles in the paraffin matrix, respectively [15]. The incorporation of aluminum foils with the paraffin PCM marginally improved the conductivity by 0.63 W/mK. Moreover, the aluminum foam with paraffin showed better discharging results, making them a promising material to be incorporated in a Li-ion battery system [21]. Steatite, often known as soapstone, is a magnesium-rich metamorphic rock comprised primarily of the mineral talc. Steatite is a chemical compound with the formula MgSiO_3_. In addition, steatite is a prominent material for making operational products across the world, such as paints, ceramics, cooking pots, woodstoves, electrical panels, kitchen surfaces, and molds for metal casting, because of its economic relevance [22,23].

Based on the broad literature survey, this research aims to fabricate a PCM/steatite composite and study the effect of the cyclic load on the paraffin PCM encapsulated with and without ball-milled steatite on a horizontal LHTES system. The thermal stability of steatite is excellent (usually greater than 1000 °C), and the present experimental investigation is mainly focused on the thermal characteristics study of the composites. The effects of steatite (milled and not milled) on the microstructure PCM composites were examined. All characterization investigations were performed to reveal the successful incorporation of PCM/steatite composite and its properties. To the best of our knowledge, PCM/steatite composites have not been fabricated, and information about their thermal characteristics is limited. 

## 2. Materials and Methods

### Composite Preparation

Paraffin wax (50 g) was chosen as the matrix material in the present investigation, with steatite (5 g) being the additive material in the preparation of PCM. Table 1 shows the thermophysical properties of the chosen material. The High Energy Ball Milling (HEBM) process was used to mill steatite powder, and Retsch PM100 planetary type (Sunpro International Group, Taipei City, Taiwan) machine was used for ball milling. To have better milling, a ball to powder ratio of 12:1 in an argon gas atmosphere was maintained. The rotation speed was 300 rpm for 18 h of milling time. A hot plate was used to melt 50 g of paraffin wax to 70 °C. Then, 5 g of steatite powder was mixed with melted paraffin and stirred at 500 rpm for one hour. The mixtures were then poured into the Petri plates and cooled at room temperature to make a PCM composite. Similarly, the same process was carried out for the ball-milled steatite powder. Energy storage material with different compositions and abbreviation are shown in Table 2.

**Figure 1:**
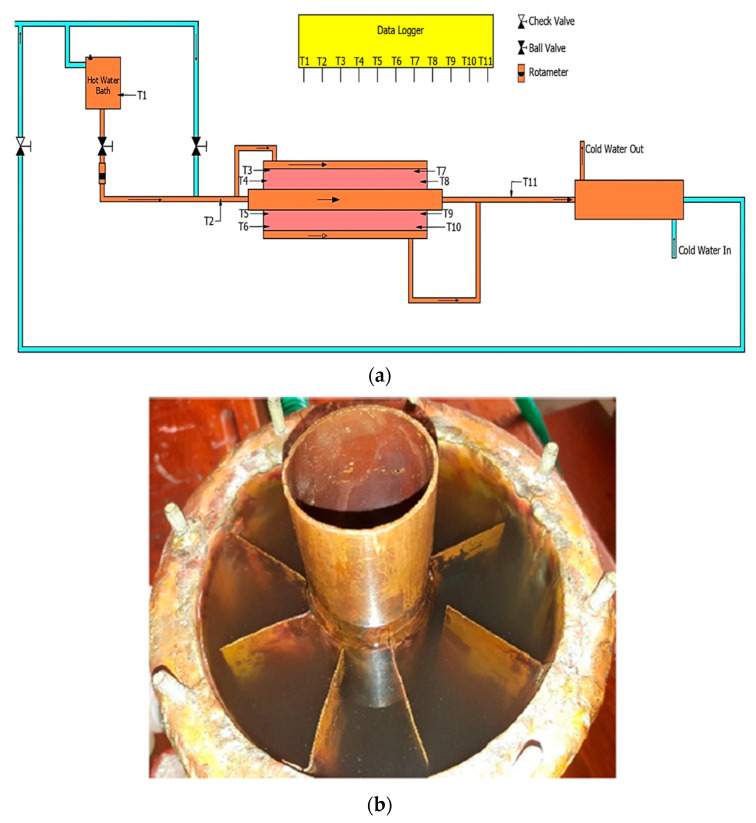
(**a**) Schematic of LHTES setup. (**b**) Schematic of fin arrangement.

The rate at which the PCM material charged and discharged was measured using a horizontal latent heat TES system (Figure 1), which was made in accordance with Abduljalil et al. [16]. The test chamber was composed of three copper concentric tubes through which the heat transfer fluid (HTF) flows The paraffin/steatite composite was filled into the intermediate tube of the three concentric tubes. The HTF and paraffin/steatite composite were increased by attaching longitudinal fins of 480 mm length, 42 mm width, and 0.8 mm thickness. The composite in this test section was measured using eight K-type thermocouples. Table 3 presents the location of each probe. The entire test readings were recorded using a data logger system, and 50 mm glass wool was used to reduce heat loss.

Table 4 presents the charging and discharging test parameters. The readings were recorded with an equal time interval of 15 min. Three trials were conducted under each testing condition to analyze the thermal behavior of the paraffin–steatite PCM. X-ray diffraction (XRD) analysis was used to determine the phases of the steatite. A scanning electron microscope (SEM) (model JSM-6390LV, JEOL USA Inc., Peabody, MA, USA) equipped with an EDS was used to examine the microstructures of PCM composites.

## 3. Results and Discussion

### 3.1. XRD Analysis

**Figure 2:**
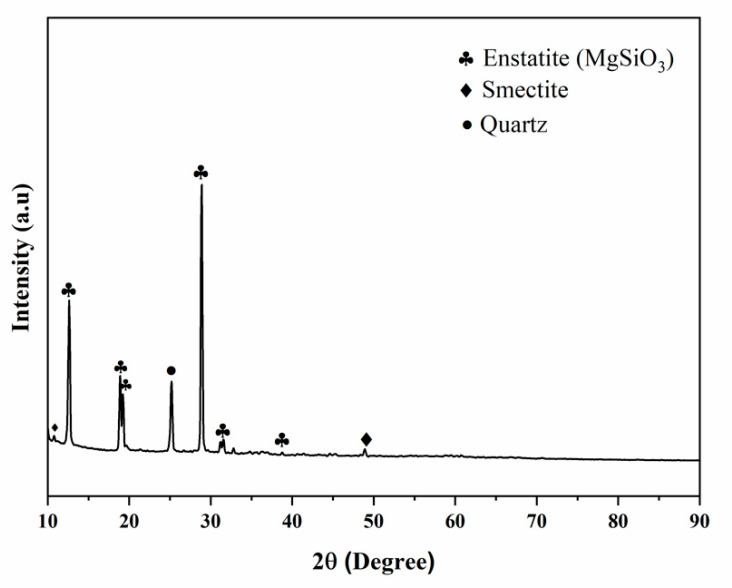
XRD pattern of the composites.

Figure 2 depicts the XRD examinations of the steatite powder sample phase. Some same phases were seen in the XRD analysis of steatite powders; these included the enstatite (JCPDS 86-0430), quartz (JCPDS 46-1045), and smectite families (JCPDS 13-0135). In an XRD analysis, the peaks were mostly found between 10° and 30°. A mineral phase known as enstatite (MgSiO_3_) was derived from the talc abundant in steatite powder [24].

### 3.2. Particle Size Analysis

**Figure 3:**
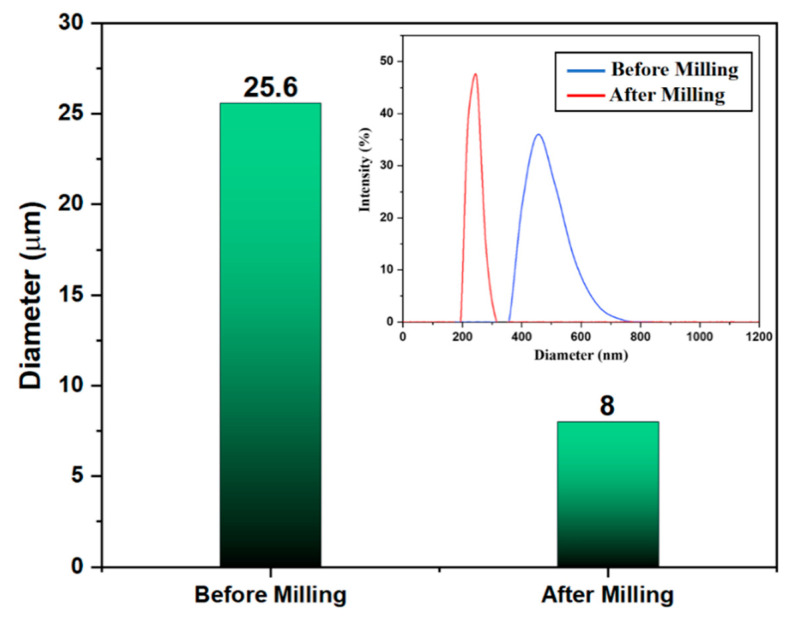
Particle size measurement.

**Figure 4:**
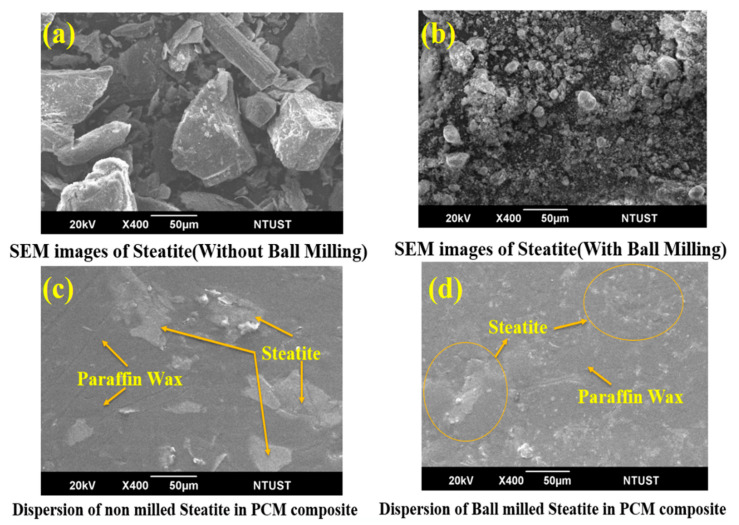
SEM images of (**a**) steatite powder (without milling), (**b**) steatite powder (after milling), (**c**) PCMS composite, and (**d**) PCMSB composite.

A laser diffraction method was used to evaluate the average sizes of the particle and particle size distributions prior to the experiment. Figure 3 shows the average steatite particle sizes obtained before and after 18 h of milling time. Dalmis et al. noted that when the milling times exceeded 8 h, the structure was unable to handle the increased stress and plastic deformation, and thus, the particle broke into smaller particles. Figure 4b also showed that the steatite particle size was reduced to a greater extent after ball milling. The ball milling process also improved the steatite wettability and distribution within the PCM composite, thereby improving the PCM composite characteristics Figure 4d.

### 3.3. Microstructural Study

Figure 4 depicts the steatite powder morphology before and after milling. The steatite powder before milling exhibited rod-, plate-, or flake-shaped particles with varying sizes, as shown in Figure 4a. Given the different shapes and sizes of the particle, Figure 4c shows the nonuniform dispersion of the steatite particles in the PCM composite. The steatite powders were then ball milled for 18 h. During milling, the ball stress on steatite particles increases, and the particles cannot withstand their structure. As a result of the milling, the particles are deformed into small and fine particles, as shown in Figure 4b. The effective stirring and smaller size of the milled steatite particles cause uniform dispersion, and they exhibit good physical interaction with the paraffin wax without agglomeration in the composite, Figure 4d. Moreover, no alteration occurred in the layers of the paraffin, and instead, the nanoparticles were diffused inside the matrix. A good dispersion of the milled steatite in the paraffin wax facilitated a good connecting network and resulted in an enhancement of the heat transfer [25].

### 3.4. DSC Analysis 

**Figure 5:**
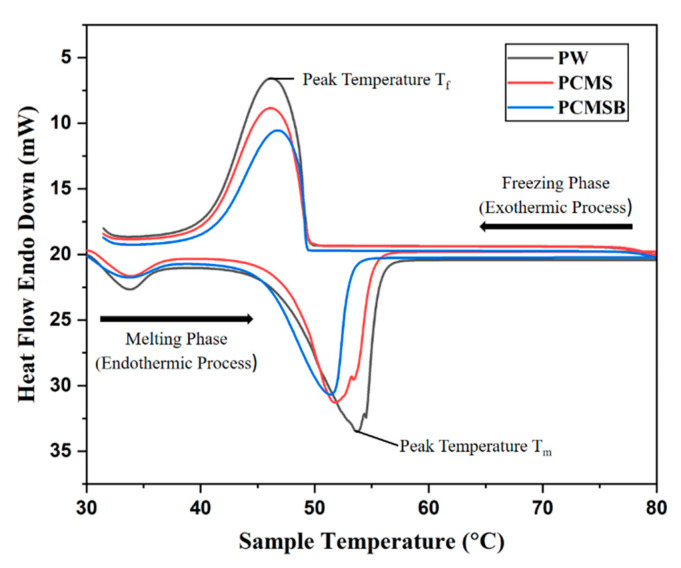
DSC curves of the paraffin wax/steatite composite.

A differential scanning calorimeter (DSC 6000, PerkinElmer, Inc., Waltham, MA, USA) was used to examine the thermal characteristics of the PW, PCMS, and PCMSB. During melting, a PCM composite sample was heated continuously from 30 °C to 80 °C at a rate of 5 °C/min and then cooled back to 30 °C. Figure 5 shows the heating and cooling behavior curves of the paraffin wax and PCM/Steatite composites.

In the DSC curves, two significant transition peaks could be seen through the heating process. The sharpest peak corresponded to the solid–liquid phase change of the paraffin wax, whereas the minor peak corresponded to the solid–solid phase change. Storage systems with solid-to-solid phase changes have low phase change enthalpies and are characterized by small volume changes compared with systems with solid-to-liquid phase changes [26]. From Table 5, we see that the peak melting temperature (Tpeak-m) of the PCMS and PCMSB composite were lower than those of pure paraffin at 2.4 °C and 3.1 °C, respectively. There was also a 1.8 °C difference in the melting temperature (Tm) between the pure paraffin wax and the PCMSB composite. Because of the change in steatite particle size due to the ball milling, they tend to have a lower melting temperature. The results showed that the ball-milled steatite acted as a nucleation agent, helping to increase crystallization, and thus reducing the supercooling effects of PW. For any latent heat storage system, a stable phase transition assisted by reduced super cooling is needed to attain better stability in the discharging process. In the present case, the presence of steatite helped to gain better phase stability [24]. The low melting temperature implied that the PCMSB composite absorbed more heat from the HTF than PW, which was due to the higher thermal conductivity of the steatite. In addition, the ball-milled steatite reduced the thermal resistance of melting and solidification by steadying the temperature variation.

The Maxwell–Garnett equation [25] was used to calculate the thermal conductivity of PCMSB composites.

$$K_{Pcm}=K_{p}\left[\frac{K_{s}+2K_{p}+2\emptyset \left(K_{s}-K_{p}\right)}{K_{s}+2K_{p}+\emptyset \left(K_{s}-K_{p}\right)}\right]$$

where ∅ is the volumetric ratio, *p* is the paraffin wax, and *s* is the steatite material.

Based on the thermal conductivity calculation in the above equation, it is estimated that both the thermal conductivity of PCMS and PCMSB were in the same range theoretically as the volumetric ratio remained the same. However, the thermophysical properties differed due to the high-energy ball milling process conferred by the DSC results. With the increasing mass fraction of the steatite powder, the PCMSB composites exhibited higher thermal conductivity. The composites composed of PCMSB had an average thermal conductivity of 0.2226 W/mK. In comparison with paraffin, the thermal conductivity value of the PCMSB composite was 7.7% higher. The enthalpy value of PCMSB seemed to increase when compared to the PCMS, and for the ball-milled composites, the value also seemed to marginally increase the latent heat capacity. The solid PCM/steatite composite received heat initially, leading to an increase in temperature over time. In the following step, a melting process began and continued until the solid PCM composite transformed into the liquid state. As the PCM composite moved into the melting zone, its temperature increased slightly. Due to the continuous heat supply during melting, the PCM composites had a higher latent heat value. In order to begin the solidification process, the liquid PCM must cool down. During the solidification of the PCM, the temperature was nearly constant because high heat transfers through convection. The solidification of the PCM composites followed the removal of heat from the liquid PCM. Due to the heat being released, the latent heat of freezing was lower [26]. As a result, the addition of the steatite powder to the paraffin wax resulted in significant changes in the thermal properties of the PCMS and PCMSB composites.

### 3.5. Performance during Charging and Discharging

**Figure 6:**
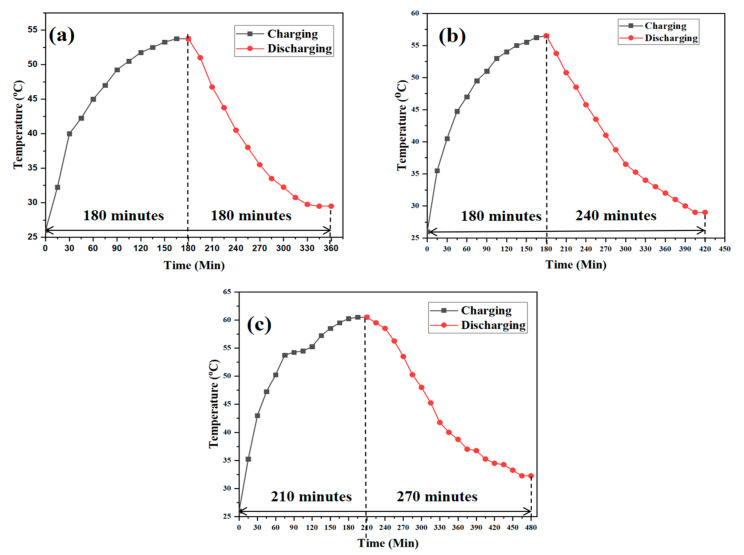
Temperature curves of the paraffin/steatite composite during the charging and discharging phase: (**a**) PW, (**b**) PCMS, and (**c**) PCMSB.

During the charging and discharging processes, the data logging system was used to determine the average temperature of the PCMS and PCMSB composite. Heat is transferred more efficiently through longitudinal fins. Temperatures were recorded based on three different trials, and the average readings were calculated. Figure 6 shows the average experimental values measured on the LHTES system. Pure paraffin PCM (Figure 6a) took 180 min for the complete charging operation, whereas the PCMS (Figure 6b) and PCMSB (Figure 6c) exhibited 180 and 210 min to attain a steady state. The increase in the thermophysical properties of the composites and the influence of the mechanical milling increased the charging time indicating the greater amount of thermal energy being stored. The graphs show that the discharging time of the PCMSB was greater than the steatite encapsulated PCM. The PCMSB took 270 min to attain the steady state against the 240 min recorded for PCMS, thereby indicating the increase in thermal stability and operational sustainability during the discharging process. The use of ball-milled steatite additives that were highly thermally conductive in PCM composites enhanced the thermal conductivity and increased the heat transfer efficiency. During the transition from liquid to solid, the material experienced supercooling. Since the crystallization process was delayed, the material remained liquid even at cold temperatures. Consequently, the composite with ball-milled steatite fabricated composite exhibited better discharge characteristics [27]. Figure 6a–c, which denote the experimentation data using the PCM, PCMS, and PCMSB systems, show a significant increase in the available latent heat discharging time. The addition of steatite had only a marginal increase in the latent heat capability in the case of both PCMS and PCMSB. The enhancement in the discharge time was correlated with the better stability attained due to a reduction in particle size. The increase in melting and solidification time was due to the smaller particle and increased thermal conductivity of the ball-milled steatite. The thermal conductivity of the composites depended on the bonding between the atoms. The higher the bonding, the greater the melting temperature, and the thermal conductivity will also be higher, as the heat flow will be higher in these substances. This remains the reason behind the increase in melting time with respect to the increase in the thermal conductivity. Moreover, conduction was the major heat transfer mode occurring across the system during the discharging process. On average, the melting time increased by 17% with the PCMSB composite, compared with the pure paraffin and PCMS. The increase in the liquefaction period denoted the enhanced capability to utilize the available sensible heat. The discharging cycle results showed an increase in the solidification time by incorporating ball-milled steatite with paraffin PCM. This enhancement denoted the capability of the PCM to utilize the available latent heat to store and discharge with an enhanced time duration.

## 4. Conclusions

The results obtained by experimentally studying the melting and freezing of the paraffin/steatite composite led to the following conclusions:

- Incorporating steatite powder with paraffin wax enhanced the heat transfer of the energy storage system, consequently increasing the rate of charging and discharging.
- The usage of the ball-milled steatite composite increased the discharging time at 50% and the micro-size steatite composite at 33.33%. The thermal conductivity value of the milled steatite-based PCM composite was 7.7% higher than PW.
- The overall increase in the discharge time showed the capability of the PCM material to store the available thermal energy and discharge it as needed by the incorporated system.
- PCM reinforcement with ball-milled steatite in solar water heaters enhances the storage of heat energy and provides hot water availability for a longer period at a higher temperature.

## Figures and Tables

**Table 1:** Thermophysical properties of PCM materials.

Properties	Paraffin Wax	Steatite
Density (g/cm^3^)	0.861	2.7
Specific Heat (KJ/kg K)	2.8	0.921
Thermal conductivity (W/mK)	0.214	2.9
Melting Point (°C)	56	1600

**Table 2:** Energy storage material with different compositions.

Name	Composition
PW	Paraffin Wax
PCMS	Paraffin Wax/Steatite Composite
PCMSB	Paraffin Wax/Ball-milled Steatite Composite

**Table 3:** Probe location.

Thermocouple Probe	Probe Location
T3, T6 T7, T10	150 mm from either side of the test section
T4, T5,T8, T9	50 mm from either side of the test section

**Table 4:** Experimental conditions during the charging and discharging cycles.

Condition	Fluid Flow Rate
Charging	Hot fluid flow rate = 0.0055 kg/s
	Hot fluid temperature = 60 °C
Discharging	Cold fluid flow rate = 0.0033 kg/s
	Cold fluid temperature = 30 °C

**Table 5:** Thermophysical properties obtained from the DSC analysis.

Sample	The Melting Process			The Solidification Process		
	*T*_m_ (℃)	*T*_peak-m_ (℃)	*H*_m_ (J⋅g^−1^)	*T*_m_ (℃)	*T*_peak-m_ (℃)	*H*_m_ (J⋅g^−1^)
PW	47.2	53.7	163.5	49.3	46.3	165.2
PCMS	46.6	51.4	133.8	48.9	46.0	134.5
PCMSB	45.4	50.7	135.2	46.2	44.8	132.47

## Data Availability

No data availability statement.
